# Emotion Unchained: Facial Expression Modulates Gaze Cueing under Cognitive Load

**DOI:** 10.1371/journal.pone.0168111

**Published:** 2016-12-13

**Authors:** Anna Pecchinenda, Manuel Petrucci

**Affiliations:** Department of Psychology, Sapienza University of Rome, Rome, Italy; University of Bologna, ITALY

## Abstract

Direction of eye gaze cues spatial attention, and typically this cueing effect is not modulated by the expression of a face unless top-down processes are explicitly or implicitly involved. To investigate the role of cognitive control on gaze cueing by emotional faces, participants performed a gaze cueing task with happy, angry, or neutral faces under high (i.e., counting backward by 7) or low cognitive load (i.e., counting forward by 2). Results show that high cognitive load enhances gaze cueing effects for angry facial expressions. In addition, cognitive load reduces gaze cueing for neutral faces, whereas happy facial expressions and gaze affected object preferences regardless of load. This evidence clearly indicates a differential role of cognitive control in processing gaze direction and facial expression, suggesting that under typical conditions, when we shift attention based on social cues from another person, cognitive control processes are used to reduce interference from emotional information.

## Introduction

The human face is a rich source of information: we are sensitive to where another individual is looking, and by shifting eye gaze to the same location, we have a good idea of their focus of interest [1]. Similarly, we use the facial expression of another individual to draw inferences about their emotions, we process facial expressions even when they are to be ignored [2, 3], and we automatically interpret changes in facial expressions as reflecting changes in mental states [4], although there is also evidence that processing emotional faces can be resource-dependent [5, 6]. Over the past years, researchers have investigated whether the information conveyed by eye-gaze and the facial expression of another individual affects where we look using the gaze cueing paradigm [7]. This is a variant of the standard attentional cueing paradigm, in which the central symbolic cue is replaced by a face gazing left or right and participants respond as quickly as possible to a peripheral target appearing shortly after the non-predictive gaze cue. Typical findings consist in faster responses to targets presented at the spatial location looked at by the face (i.e., valid cue) than to targets presented at the opposite spatial location (i.e., invalid cue). To date, two questions concerning gaze cueing effects are still open to investigation: 1) whether gaze cueing effects are modulated by faces showing an emotional expression and 2) to what extent gaze cueing effects elicited by unpredictive, central gaze cues shares characteristics of exogenous, reflexive, automatic attention (for a review see Chica et al. [8]).

### Modulation of Gaze Cueing Effects by Facial Expression

Research on whether gaze direction and facial expression interact in orienting attention has provided a mixed picture. Findings have typically shown that gaze cueing effects are independent of facial expression [9–14]. That is, although the emotional expression would provide useful information to the observer as to what another person is looking at, gaze cueing effects are not modulated by whether the face shows an emotional or a neutral expression. In contrast, a growing number of studies has reported greater gaze cueing effects when faces show an emotional expression. Enhanced gaze cueing effects have been observed for fearful expressions but only for anxious participants [15, 16], whereas other researchers have reported enhanced gaze cueing effects for fearful and surprised faces but not for angry or happy faces [17–19]. However, larger gaze cueing for fearful and surprised expressions have been attributed to the role of low level perceptual features around the eyes such as the wide open eyes and the greater sclera/pupil contrast [20]. Enhanced gaze cueing effects for happy faces have also been reported [21].

Interestingly, evidence also shows that in the gaze cueing paradigm, the emotional expression is processed as it affects individuals' preferences toward objects used as targets. Namely, Bayliss et al. [10] used images of kitchen and garage objects as targets in a gaze cueing task with happy and disgusted faces. Participants completed two blocks of gaze cueing trials and in the third and last block they were also asked to rate their preference toward the targets. Findings showed that objects presented with happy faces on valid trials (i.e., happy face gazing at the object) were preferred to objects presented with disgusted faces.

Attempts to explain these different findings have suggested that emotional expressions may hold attention resulting in overall slower responses, rather than in faster shifts of attention in the direction of eye gaze and away from the face [22]. However, such an account would yield null gaze cueing effects, particularly for threat expressions, which are known to attract and hold attention [23]. Alternative accounts for the enhancement of gaze cueing effects by the emotional expression of the central face-cue have called upon the role of top-down factors, such as instructions to adopt an evaluative goal [11], priming individuals with threat pictures before the gaze cueing task [24], or providing an emotional context by using emotional targets [25–27]. However, even in this latter case, findings are not straightforward as in some studies contextual effects have not been observed. Namely, contextual effects on gaze cueing have been observed with infants and children but not with adults [28–29].

Yet another account points out, based on ERP data, that the emotional expression of a face is processed too quickly (as indexed by modulation of P135) compared to the direction of eye gaze (as indexed by modulation of N190) for both information to simultaneously affect attentional shifts [30]; but see [31] for evidence of attention modulation by gaze direction already at 55–70 ms post-stimulus when using MEPs, a more time-sensitive measure of brain activity. Consequently, a series of studies has systematically varied the timing between the onset of gaze shifts and the onset of changes in emotional expression (i.e., the central face first shifts gaze direction and then changes expression from neutral to emotional). Whereas in these cases, evidence shows that emotional expressions modulate gaze cueing effects, it is unclear why in Bayliss et al. [10] who used similar manipulations (i.e., changes in gaze shifts *followed* by changes in expression) emotional expressions did not modulate gaze cueing effects despite they affected objects' preferences. Interestingly, this account taps also on the second issue still open to research: whether gaze cueing shares characteristics of exogenous, automatic attention.

### Characteristics of Orienting by Gaze Cues

In fact, because of the social and biological importance of the eyes, gaze cueing was originally considered a special case of reflexive orienting by a central cue, although similar cueing effects have also been demonstrated with unpredictive, central arrow-cues [32, 33]. To determine to what extent attentional shifts by unpredictive, central gaze cues involve exogenous or endogenous attention, researchers have used a concurrent task, loading on working memory resources. This is because endogenous attentions is affected by demands for cognitive resources whereas exogenous attention is not [34]. Indeed, Law, Langton, and Logie [35] asked participants to perform a gaze cueing task with neutral faces presented centrally. Gaze direction was not predictive of target location and participants performed this task concurrently to a verbal (exp. 1) or visuospatial (exp. 2) working memory task. Findings showed that gaze cueing effects were unaffected by either verbal or visuospatial load. More recently, Hayward and Ristic [36] asked participants to perform a gaze cueing task under verbal working memory load and manipulated whether gaze direction was counterpredictive (exp. 1) or predictive (exp. 2) of target location. When gaze direction is counterpredictive, shifts of attention can be based on voluntary, top-down processes linked to the meaning attributed to gaze-cue, tapping onto endogenous attention (i.e., gaze looking to the left means target appearing to the right), or they can be driven by gaze-direction itself, tapping onto the more involuntary and reflexive component of attention. Findings showed that when orienting was based on the effective direction of eye gaze, gaze cueing effects were unaffected by working memory load. In contrast, when gaze direction was counterpredictive of target location and diverged spatially from orienting by gaze direction, gaze cueing effects were affected by working memory load. In contrast, Bobak and Langton [37], arguing on the importance of the type of load used, have recently used neutral faces and showed that gaze cueing effects by unpredictive central gaze cues are disrupted when participants concurrently perform a random generation task, which loads on cognitive control and executive functions, but they are not affected by a task that requires simply to repeat an overlearned number sequence (1 to 9). However, in this study, participants performed the random number generation at a fixed external pace, which may have affected response timing to the target (i.e., responding as fast as possible to a target may be subordinate to keeping with the external pace). In fact, in this study also orienting by peripheral exogenous cues (i.e., changes in luminance), which is deemed to be reflexive, was reduced when participants performed the random number generation at a fixed external pace.

To summarize, although gaze direction and emotional expression are both present on a face at any given time, and they can be processed automatically, it is still unclear why they are not combined in affecting attentional shifts as assessed by gaze cueing effects as they may provide useful information on what attracts the other person's interest. Instead, facial expression modulates gaze cueing effects only under certain conditions. A possible way to reconcile this body of evidence points to the involvement of cognitive control processes in resolving the conflict between allocating attention to task-irrelevant, but emotionally salient distractors and maintaining task priorities. Accordingly, task irrelevant emotional expressions would not modulate gaze cueing effects because participants use cognitive control to reduce their interference. Indeed, recent evidence points to this direction as Holmes, Mogg, de Fockert, Nielsen, and Bradley [38] used a dot-probe task with pairs of faces (neutral and angry) and a working memory load consisting in retaining a series of digits in numerical (low load) or random order (high load). Typical results reveal faster responses to probes presented at the location previously occupied by the task-irrelevant angry face (i.e., attentional bias for angry faces). Findings showed that high working memory load enhanced the attentional bias toward angry distractor-faces, suggesting that, under typical circumstances, cognitive control is used to comply with instructions to ignore the two peripherally presented faces, as they are not predictive of where the probe appears. However, when cognitive control processes are taxed by a concurrent task, this priority cannot be efficiently maintained and attention is captured by the peripherally presented emotional face. This interpretation calls upon a less all-or-none conceptualization of automaticity and is also in line with evidence on the interplay between exogenous, emotional, and endogenous components of attention in the dot-probe task [39]. In a similar vein, albeit processing social cues can be automatic, it can also be affected by top-down factors, therefore one may argue that when emotional faces gazing left or right are used as uninformative central cues in the gaze cueing task, cognitive control processes are engaged to comply with task instructions and maintain task priority. If this were the case, this selective process should be less efficient when cognitive control is taxed by a concurrent task. To our knowledge, it has not been investigated to what extent loading cognitive control mechanisms affects the interplay between task priority and stimulus emotional salience in the gaze cueing task.

The present study investigated the role of cognitive load on gaze cueing effects with emotional faces. We reasoned that, if gaze cueing is not modulated by the emotional expression of a face because cognitive control is used to ignore emotionally salient distractors, then using a task that heavily loads cognitive control should hinder maintaining task priority and result in gaze cueing effects being modulated by the emotional expression. To this aim, participants performed a gaze cueing task with emotional and neutral faces while counting aloud throughout the task. For one group, cognitive load was high (counting backwards in steps of 7), whereas for the other group, cognitive load was low (counting forward in steps of 2). We chose to use counting forwards and counting backwards to manipulate load because both tasks involve similar underlying operations (i.e., constant monitoring, maintenance, manipulation, and updating of information) that rely on cognitive control processes and executive functions, albeit counting forwards in steps of 2 is much less demanding than counting backward in steps of 7 [40, 41]. We used this task as loading working memory may not be sufficient to tax cognitive control resources [42] and because we have successfully used it to load cognitive control [43]. In addition, we chose to use a gaze cueing task with neutral and emotional expressions and an SOA of 250 ms typical of automatic effects [44] and because optimal SOA duration for gaze cueing effects has been reported to be <300 ms [10, 12, 25, 26, 35, 37], and gaze cueing effects disappear at longer SOA [26]; for a review see [45], which taps on the issue of gaze cueing effects having characteristics of automaticity. If emotional expression and gaze direction are processed automatically, they should not be affected by cognitive load when using parameters that allow investigating for automatic effects. Furthermore, by including neutral expressions, the present study also contributes to clarifying whether cueing effects by central unpredictive gaze-cues depend on cognitive control resources being available. We used happy and angry expressions as negative and positive emotions associated with approach motivation [46] although angry expressions are also threat signals without the characteristic wide opened eyes of fearful expressions [20]. In addition, from a dimensional approach, the major difference between happy and angry faces is on the valence dimension rather than on the arousal dimension [47, 48]. Accordingly, recent evidence shows that adults rate happy and angry faces very similarly on the arousal dimension [49]. Finally, we used the same objects as targets as in Bayliss et al. [10] and collected preference ratings in the third block to assess whether emotional expression and gaze direction affect object preferences even when faces concurrently change gaze direction and emotional expression under cognitive load.

To preview our findings, cognitive load engendered larger gaze cueing effects by angry emotional expressions and disrupted gaze cueing effects by neutral faces. In addition, happy facial expressions and gaze affected object preferences regardless of load.

## Method

### Participants

Seventy-seven participants (60 females, 17 males, age *M* = 21.6; *SD* = 2.2 completed the experiment in partial fulfilment of course credits. Thirty-nine participants were randomly assigned to the high cognitive load (29 females, 10 males, age *M* = 21.9; *SD* = 2.3) and 38 (31 females, 7 males, age *M* = 21.3; *SD* = 2.0) to the low cognitive load group. They all had normal or corrected to normal vision and were naïve to the experimental hypotheses. All participants gave their written informed consent, which was obtained according to the Declaration of Helsinki (1991). The experiment was in compliance with institutional guidelines and had received approval by the Department of Psychology Ethics Committee, Sapienza University.

The data from additional 5 participants were excluded as scoring of the recorded counting task revealed they did not comply with the instructions of counting aloud throughout the entire gaze cueing task.

### Materials and Apparatus

Four faces (2 female and 2 male: BF06, BF11, BM10, BM11) displaying a happy, angry, or a neutral expression were selected from the Karolinska Directed Emotional Faces set [50]. All faces had straight eye-gaze and for each face, two versions were created using Photoshop: one gazing left and one gazing right. Target stimuli were as in Bayliss et al. [10] and consisted of 36 images of household items: 18 belonged to the "garage" category (i.e., screwdriver), and 18 belonged to the "kitchen" category (i.e., teapot). Two versions of each image were used, one oriented to the left and one to the right. All stimuli were converted to gray-scale. The gaze-cueing task was presented using E-Prime Version 2.0 Professional software [51] for Windows 7, which also recorded participants’ responses. Stimuli were presented on a Pentium IV computer via a 17” CRT monitor (1024 x 768 pixels, 60 Hz). Responses were entered using a standard USB-keyboard with timing error less than 1 ms.

### Procedure

After participants had given informed written consent, they sat in front of a computer in a dimly lit room and read the instructions presented on screen. The experiment consisted in performing two tasks simultaneously: the gaze cueing task and counting aloud.

For the gaze cueing task, participants completed 26 practice trials, followed by 648 trials divided in 3 blocks of 216 trials. Each block consisted of 192 experimental trials with equally probable factorial combination of facial expression (happy, neutral, angry), gaze direction (left, right), target position (left, right), and target category (kitchen, garage) and 24 catch trials (4 for each facial expression gazing left and 4 for each facial expression gazing right). Assignment of target category to male and female face-cues was balanced within blocks, whereas assignment of target-objects to emotional face-cues was counterbalanced in different versions of the task, so that if an object was assigned to happy valid faces in one version of the task, it was assigned to angry valid faces and to neutral valid faces in the other versions. For all trials, the direction of eye gaze was equally likely to look toward (i.e., valid cue) or away from (i.e., invalid cue) the target.

For the counting aloud task, at the beginning of each block a 4-digit number (i.e., 1216 in the practice block, 4564, 4653 and 5216 in Block 1, 2, 3 respectively) appeared on screen with the instructions to start to count aloud, backward in steps of 7 (high cognitive load group) or forward in steps of 2 (low cognitive load group). To stress the importance of the counting task, at the end of each block participants were prompted, by instructions presented on screen, to type the number they had reached counting. The need to perform the two tasks in parallel and to prioritize both was strongly emphasized and verified during the practice session. Participants were informed that counting aloud was recorded using a digital recorder for later scoring and they first practiced counting alone, then the two tasks combined. During the practice trials, the experimenter assisted participants with the dual task procedure, answered any questions they had and ensured it was clear they were required to continue to count aloud while performing the gaze cueing task.

**Fig 1:**
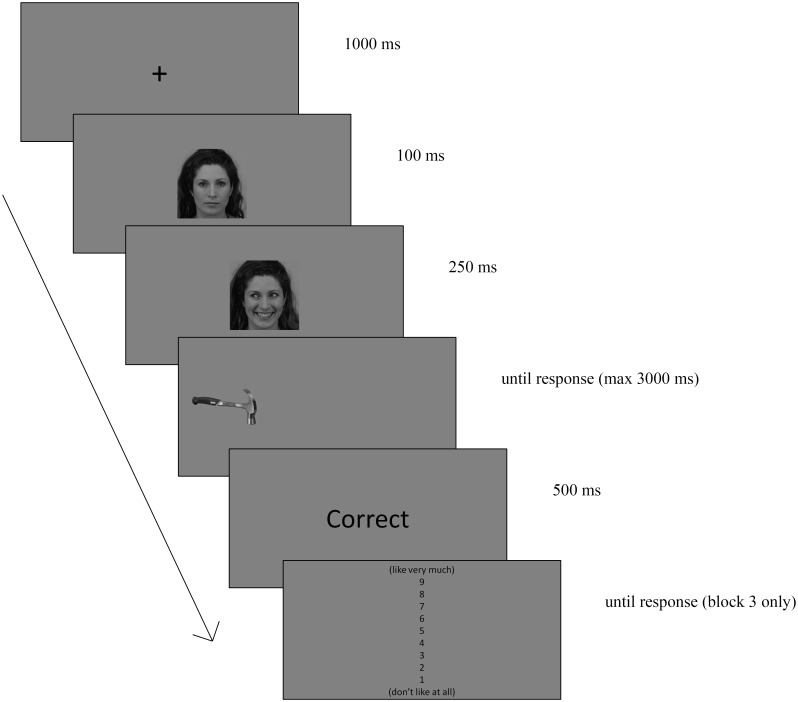
Examples of stimuli and sequence of events used in the gaze cueing task in block 3 (KDEF image id: BF11HAS).

Each trial started with a fixation cross for 1000 ms, followed by a neutral face looking straight ahead for 100 ms, which signalled the central face-cue appearance. The same face was then presented for 250 ms but this time, the expression could be happy, angry or remain neutral and it could look left or right. A target would then appear to the left or right of the face and remained on screen until response or 3000 ms had elapsed. At a viewing distance of 60 cm, central face-cues subtended 18.0° by 11.2° of visual angle and target-objects subtended 7.1° by 2.4° of visual angle, whereas the center-to-center distance between central face-cues and targets was 11.2° of visual angle. For the catch trials no target was presented. A response feedback ("Correct" or "Wrong") followed for 500 ms. The ITI was 500 ms (see Fig 1).

As in Bayliss et al. [10], in the third block the procedure changed slightly and participants were informed that after responding to the target, the question ‘‘How much did you like that object?” would appear at the top of the screen with a 9-point scale, in which 9 indicated ‘‘Like very much,” and 1 indicated ‘‘Don’t like at all”. Their task was to rate the target-object by pressing the selected number (1–9) on the keyboard using the left hand.

Participants were informed that gaze direction was not predictive of target position and that in some trials (catch trials) no target would appear. They were instructed to respond only when a probe was presented based on its location as quickly and accurately as possible and to continue to count aloud throughout the task. Participants responded by pressing the keys "u" and "b" of the keyboard labeled "left" and "right", chosen to be perpendicular to the left/right target position. Key assignment was counterbalanced between participants.

### Experimental Design

The experimental design was a 3 (Facial Expression: Happy, Angry, Neutral) by 2 (Gaze Direction: Left *vs*. Right) by 2 (Target Position: Left *vs*. Right) by 2 (Cognitive Load: High *vs*. Low) mixed-factorial with the last factor between-subjects.

### Data Analyses

#### Counting task

Scoring of the recorded counting aloud task revealed that participants counted aloud throughout the entire task. Participants who counted backward in steps of 7, performed an average of 135 operations (*SD* = 50), with an overall accuracy of 89%, (*SD* = 7.6). Participants who counted forward in steps of 2 performed an average of 447 operations (*SD* = 105), with an overall accuracy of 99%, (*SD* = .72).

#### Gaze cueing task

Trials in which an error was made (11% for the high cognitive load group and 6% for the low cognitive load group) and with RTs faster than 120 ms or 2.5 SD above the mean (8% for the high cognitive load group and 6% for the low cognitive load group) were excluded from analyses. Mean RTs and proportion of correct responses (i.e., accuracy) were computed for each condition. RTs and arcsin transformed accuracy data (untransformed mean values are reported in describing effects) were analysed with a 3 (Facial Expression: Happy, Angry, Neutral) by 2 (Gaze Cue: Valid *vs*. Invalid) by 2 (Cognitive Load: High *vs*. Low) mixed-factorial ANOVA.

## Results

**RTs:** Means and standard errors for all conditions are reported in Table 1.

**Table 1:** Mean RTs (SE) as a function of Facial Expression and Cue Validity for the Low Cognitive Load and High Cognitive Load.

Cognitive Load	Expression	Valid Cue	Invalid Cue
Low	Angry	612 (17)	637 (27)
	Happy	602 (17)	615 (18)
	Neutral	614 (18)	619 (17)
High	Angry	705 (17)	773 (26)
	Happy	697 (17)	707 (18)
	Neutral	709 (18)	719 (16)

ANOVA results for RTs showed a significant main effect of Cognitive Load, *F* (1, 75) = 15.58, *p*< .001, partial η^2^ = .172 due to longer RTs for the High Cognitive Load group (*M* = 719 ms; *SE* = 18.14) than for the Low Cognitive Load group (*M* = 617 ms; *SE* = 18.38). The main effect of Facial Expression was significant, *F* (2, 150) = 15.82, *p*< .001, partial η^2^ = .174. Pairwise comparisons showed overall longer RTs on trials with Angry Faces, *M* = 682 ms; *SE* = 15.16, compared to trials with Neutral Faces, *M* = 666 ms; *SE* = 12.04, *p* = .018, whereas RTs were faster on trials with Happy Faces, *M* = 655 ms; *SE* = 12.13 compared to trials with Neutral faces, *p* = .003. The effect of Gaze Cue was significant, *F*(1, 75) = 30.63, *p*< .001, partial η^2^ = .290, indicating faster RTs with Valid Cue, *M* = 657 ms; *SE* = 12.05 than with Invalid Cue, *M* = 678 ms; *SE* = 14.00. The Expression by Cognitive Load, *F*(2, 150) = 2.92, *p* = .057, partial η^2^ = .037 and the Cue by Cognitive Load, *F*(1, 75) = 3.64, *p* = .060, partial η^2^ = .046 interactions did not reach full statistical significance. The Expression by Cue, *F*(2, 150) = 10.86, *p*< .001, partial η^2^ = .126 was significant. Post-hoc analyses assessed gaze cueing effects for each expression. Results showed gaze cueing effects for Angry faces, *t*(76) = 4.55, *p*< .001 due to faster RTs with Valid (*M* = 659; *SE* = 13.19) than with Invalid Cues (*M* = 706; *SE* = 20.47), and for Happy faces *t*(76) = 3.17, *p*< .002 due to faster RTs with Valid (*M* = 650; *SE* = 13.13) than with Invalid Cues (*M* = 662; *SE* = 13.50), but not for Neutral faces *t*(76) = 1.77, *p* = .081 (Valid Cue: *M* = 662; *SE* = 13.79; Invalid Cue: *M* = 670; *SE* = 12.92). This was further qualified by a significant 3-way interaction, *F*(2, 150) = 3.45, *p* = .034, partial η^2^ = .044. As an alternative analysis strategy, and to avoid the risk of over-fitting, we also compared the different models with a Bayes factor ANOVA using JASP with default prior scales [52–54]. Results showed a *BF*_*M* =_ 4.791 for the 3-way interaction model, which can be considered substantial evidence [55]. In addition, after considering all possible 2-way interaction models, the 3-way interaction model has the highest posterior probability *P(M|*data) = 0.21 and it is 1.73 times more probable (*BF*_*10*_ of the model for the 3-way interaction/*BF*_*10*_ models with 2-way interactions) than the model without 3-way interaction.

Provided substantial evidence for the 3-way interaction, we first assessed gaze cueing effects for each expression separately for each cognitive load group, by comparing RTs for Valid and Invalid cues. Results for the Low Cognitive Load group showed significant gaze cueing effects for Angry faces: there were faster RTs with Valid cues than with Invalid cues, *t*(37) = 2.63, *p* = .012. Similarly, gaze cueing effects were observed for Happy faces *t*(37) = 2.54, *p* = .015, but not for Neutral faces, *t*(37) = 1.10, *p* = .277. However, a follow-up assessed whether the lack of gaze cueing effects for the neutral faces under high as well as under low cognitive load could be due to these stimuli not changing expression but only gaze direction or to cognitive load (even low cognitive load). Participants completed the gaze cueing task only with neutral faces and no load. Findings showed significant gaze cueing effects, *F*(1, 19) = 20.82, *p*< .001, partial η^2^ = .523, Full details can be found in the Supporting Information section.

**Fig 2:**
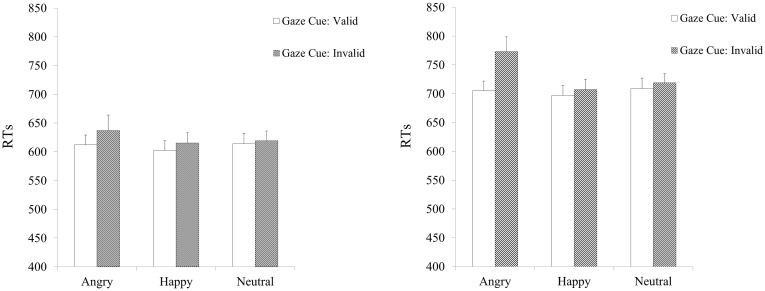
Mean RTs as a function of Facial Expression and Cue Validity for the High Cognitive Load (left) and Low Cognitive Load (right) groups. Error bars = ±1 S.E.M.

Results for the High Cognitive Load group showed gaze cueing effects for Angry faces *t* (38) = 2.63, p< .001 but not for Happy faces *t*(38) = 194, *p* = .06, which failed to reach full statistical significance, and for Neutral faces *t*(38) = 1.39, *p* = .173 (see Fig 2).

Finally, to make quantitative comparisons for gaze cueing effects between low and high cognitive load, gaze cueing indexes were computed as a percentage of overall speed [(RTInvalid—RTValid-cue)/ (RTInvalid + RTValid-cue) / 2)]*100 (see [29, 56]. Results of comparisons showed a significant difference in the gaze cueing index between Low and High Cognitive Load only for Angry faces, *t*(75) = 1.84, *p* = .035 but not for Happy, *t*(75) = .442, *p* = .33 and Neutral faces *t*(75) = .507, *p* = .30.

### Accuracy

**Fig 3:**
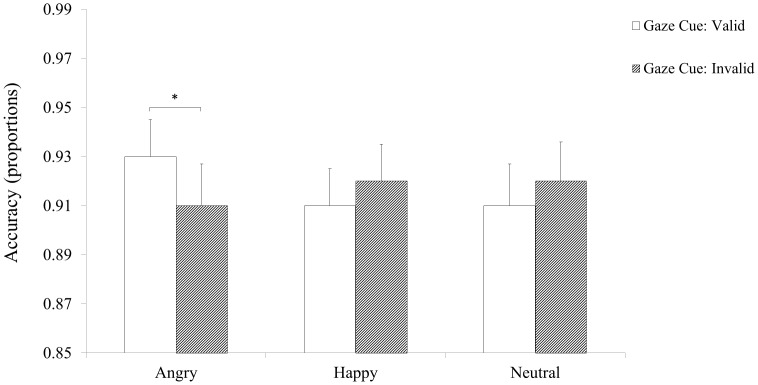
Mean accuracy to object-probe detection as a function of Facial Expression and Cue Validity for the High Cognitive Load (left) and Low Cognitive Load (right) groups. Error bars = ±1 S.E.M.

ANOVA results showed a significant main effect of Cognitive Load *F* (1, 75) = 9.25, *p* = .003, partial η^2^ = .011, due to greater accuracy for the Low Cognitive Load group (*M* = .94; *SE* = .02) than for the High Cognitive Load group, (*M* = .89; *SE* = .02). The main effects of Facial Expression *F*(2, 150) = 4.64, *p* = .01, partial η^2^ = .058 was significant: compared to trials with Neutral expressions (*M* = .91; *SE* = .01), accuracy was greater on trials with Angry (*M* = .92; *SE* = .01), *p* = .01 and Happy expressions (*M* = .92; *SE* = .01), *p* = .01. The main effect of Gaze Cue, *F*(1, 75) = .51, *p* = .476, was not significant. The Facial Expression by Cognitive Load interaction was not significant, *F*(2, 150) = .46, *p* = .63. In contrast, the Gaze Cue by Cognitive Load interaction was significant, *F*(1, 75) = 7.81, *p* = .007, partial η^2^ = .094. Post-hoc analyses showed that for both Cognitive Loads, accuracy was greater on trials with Valid Cues (Low Load: *M* = .94; *SE* = .02; High Load: *M* = .90; *SE* = .02) than with Invalid Cues (Low Load: *M* = .95; *SE* = .02; High Load: *M* = .89; *SE* = .02), *t*(37) = 19.13, *p*< .001 and *t*(38) = 20.85, *p*< .001. Accuracy differed between the two Cognitive Loads only for trials with Valid Cues, *t*(75) = 2.53, *p* = .01. The Facial Expression by Gaze Cue interaction *F*(2, 150) = 3.48, *p* = .033, partial η^2^ = .044 was significant (see Fig 3). Results of post-hoc analyses showed that, with Happy faces, accuracy did not differ between trials with Valid, *M* = .91; *SE* = .01 and Invalid Cues, *M* = .92; *SE* = .01, *t*(76) = 1.22, *p* = .23. Similarly, with Neutral faces, accuracy did not differ between trials with Valid, *M* = .91; *SE* = .01 and Invalid Cues, *M* = .92; *SE* = .01, *t*(76) = 06, *p* = .96. In contrast, with Angry faces, accuracy was greater for trials with Valid Cues, *M* = .93; *SE* = .01 compared to trials with Invalid Cues, *M* = .91; *SE* = .01, *t*(76) = 2.33, *p =* .022. Finally, the 3-way interaction was not statistically significant, *F*(2, 150) = 1.99, *p* = .82.

We also analysed accuracy data using a Generalized Estimating Equation (GEE) as an alternative analysis strategy. GEE showed no significant Expression by Cue, *Wald* (2) = 5.13, *p* = .08, Expression by Load, *Wald* (2) = 1.23, *p* = .54, Cue by Load, *Wald* (1) = 2.11, *p* = .15, and Expression by Cue by Load, *Wald* (2) = .46, *p* = .79 interactions. Therefore, both analysis strategies showed no evidence of speed-accuracy trade-off.

### Objects Preferences

**Fig 4:**
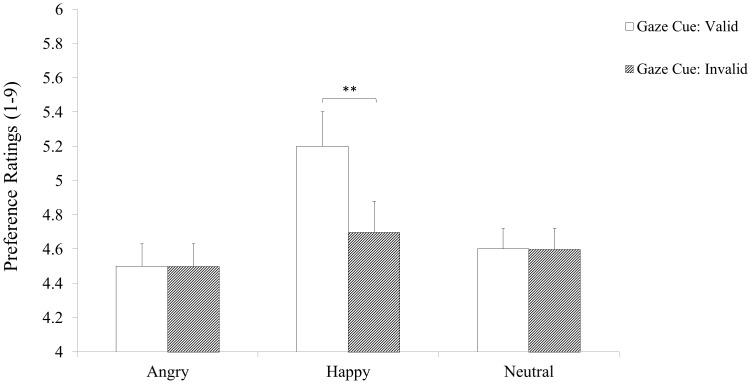
Mean object ratings as a function of Facial Expression and Cue Validity for the High Cognitive Load (left) and Low Cognitive Load (right) groups. Error bars = ±1 S.E.M.

ANOVA results for objects ratings from the last block showed that the main effect of Cognitive Load *F*(1, 75)*<* .01, *p* = .993 was not significant. There was a significant main effect of Facial Expression *F*(2, 150) = 28.43, *p*< .001, partial η^2^ = .275. Pairwise comparisons showed that object-probes presented with Happy faces *M* = 4.96; *SE* = .13, were liked more than object-probes presented with Neutral *M* = 4.61; *SE* = .12, *p* = .057 or Angry faces, *M* = 4.51; *SE* = .13, *p*< .001. The effect of Gaze Cue was significant *F*(1, 75) = 38.32, *p*< .000, partial η^2^ = .338, with higher ratings for object-probes presented with Valid Cues, *M* = 4.80; *SE* = .12 than with Invalid Cues, *M* = 4.57; *SE* = .12. The Expression by Cognitive Load, *F*(2, 150) = .267, *p* = .766, and Gaze Cue by Cognitive Load interactions, *F*(1, 75) = 1.87, *p* = .175 were not significant. However, the Expression by Gaze Cue interaction was significant, *F*(2, 150) = 14.47, *p*< .001, partial η^2^ = .162. Post-hoc analyses showed that, participants’ preference ratings were higher for target-objects presented with Happy faces and Valid Cues *M* = 5.20; *SE* = .15 compared to those presented with Happy faces and Invalid Cues *M* = 4.70; *SE* = .13, *t*(76) = 5.86, *p*< .001. In contrast, preference ratings for target-objects presented with Angry faces and Valid Cues, *M* = 4.5; *SE* = .13 did not differ from those presented with Angry faces and Invalid Cues *M* = 4.5; *SE* = .13, *t*(76) = 1.77, *p* = .080. Similarly, ratings for target-objects presented with Neutral faces and Valid Cues, *M* = 4.6; *SE* = .12 did not differ from those presented with Neutral faces and Invalid Cues *M* = 4.6; *SE* = .12, *t*(76) = 1.01, *p* = .316 (see Fig 4). Finally, the 3-way interaction, *F*(2, 150) = 1.369, *p* = .257 was not significant.

## Discussion

Research on whether gaze cueing effects are modulated by facial expressions has provided a mixed picture (see [8, 45] for reviews). Intuitively, it makes good adaptive sense that direction of eye gaze and emotional expression, two sources of information present on a face at any given time, are processed and combined together in affecting our behaviour. Yet, empirical findings show that typically, attentional shifts based on gaze direction—as assessed by gaze cueing effects—are not modulated by emotional expressions [45] unless perceptual features such as the greater sclera/pupil contrast [20] or top-down processes e.g., [11, 24–27] are involved. This is quite surprising considering that facial expressions and gaze can be processed automatically and that gaze cueing effects elicited by unpredictive, central gaze cues are thought to share characteristics of exogenous, reflexive, automatic attention, see Chica et al. [8].

The present research investigated whether, in the gaze cueing paradigm, this complex pattern of results is due to cognitive control processes being used to comply with task instructions and ignore the centrally presented, unpredictive face-cues. If this were the case, task irrelevant emotional expressions would not modulate gaze cueing effects because participants use cognitive control to reduce their interference. Participants performed a gaze cueing task under cognitive load: They either counted backward in steps of 7 (high cognitive load) or forward in steps of 2 (low cognitive load). If processing of emotional expression and gaze direction is automatic, it should not be affected by cognitive load. However, if emotional expressions are prevented from interfering with the task by cognitive control mechanisms, then cognitive load should increase the effect of emotional expressions. The results are clear: the effect of angry facial expressions on gaze cueing was enhanced by high cognitive load, whereas the effect of happy facial expressions and gaze on object preferences was unaffected by cognitive load. Interestingly, both high and low cognitive loads resulted in smaller gaze cueing effects for neutral faces. This evidence clearly indicates a differential role of cognitive control in processing gaze direction and facial expression, suggesting that under typical conditions, when we shift attention based on social cues from another person, cognitive control processes are used to reduce interference from emotional information.

In line with mood-congruent modes of processing information and allocating attention, e.g., [57], under high cognitive load angry faces increased dwell time on the invalidly cued location whereas happy faces tended to speed up responses to probe detection when validly cued by gaze and, regardless of cognitive load, happy faces affected participants' preferences toward objects validly cued by gaze [10].

To our knowledge, this is the first study showing the involvement of cognitive control processes in the gaze cueing task with emotional faces. As Bayesian analyses showed a Bayes Factor greater than 3 (BF_M_ = 4.791), the evidence of an interactive effect of cognitive load, gaze cue and facial expression can be considered substantial. By showing greater modulation by angry facial expressions of gaze cueing effects when cognitive control over emotional distractors is reduced, the present findings also shed some light on why, when the task at hand involves explicit (by task instructions) or implicit evaluative goals (by contextual effects, priming, or individual differences as when high anxiety individuals are presented with threat-related stimuli), gaze cueing effects are modulated by facial expressions. In this latter case, the conflict between maintaining task priority and processing emotional distractors is reduced (see [58, 59]) for the role of cognitive control in resolving conflict arising from task-irrelevant but emotionally salient stimuli).

The present findings also show that, gaze cueing effects for neutral faces are much reduced when participants concurrently perform a task that loads on cognitive control and executive functions. Although this is in contrast with past studies [35, 36] reporting that cueing effects by unpredictive gaze cues are unaffected by working memory load, loading working memory may not be sufficient to tax cognitive control resources [42]. Indeed, recently Bobak and Langton [37] have reported that gaze cueing effects are disrupted by a task that poses high demands on cognitive control (i.e., random number generation). The present finding for neutral faces suggests that shifting attention based on observed gaze direction might be an overlearned social skill not completely independent from cognitive resources. This evidence contributes to the rich debate on whether—and to what extent—orienting attention by social cues shares more characteristics of exogenous/reflexive or of endogenous/volitional attention. In fact, evidence that orienting attention may not be completely "reflexive" has a long history [60]. Nevertheless, this evidence would be easily reconciled by considering automaticity—included the automaticity of reflexive orienting—as a gradient, allowing different degrees of top-down modulation (for a review see [61]

Most importantly to the central question of the present investigation, the way cognitive control mechanisms are involved in modulating attention based on social cues seems to be specular for observed gaze and emotional facial expression. In fact, whereas cognitive resources are necessary to allow orienting based on gaze direction, possibly by maintaining active a goal state for being responsive to the social environment [62], cognitive control resources are used to prevent modulation of gaze-based attentional orienting by emotional facial expressions. It is interesting that in the gaze cueing task with uninformative cues, both gaze direction and emotional expression are task-irrelevant, albeit they both are salient biological stimuli, with a high potential for interference. Yet, under typical circumstances, when cognitive control resources are available, they are used to reduce interference from emotional facial expressions (i.e., ignore the expression of the central face-cue), but not from gaze direction which in fact, are necessary for being responsive to gaze (i.e., pay attention to gaze even if it is uninformative of where the target appears). It is possible that we have much more experience in ignoring facial expressions—when necessary—as we know that they can also be used to deceive. Indeed, evidence shows that the ability to process facial cues independently develops with age, as facial expressions influence the perception of gaze direction in 8-year old children but not in adults [63] and 2^nd^ and 4^th^ grade children show larger gaze cueing effects for happy compared to neutral faces [64]. Alternatively, one may argue that, albeit gaze direction is task irrelevant as it is not predictive of target position, as the “left” and “right” spatial location is specified by task instructions (i.e. respond to the target that could appear to the left or right of the face), top-down inhibition of this information would be disruptive for target detection. Therefore, preserving task performance carries along the interference from gaze direction, engendering gaze cueing effects. In contrast, when cognitive control mechanisms are taxed by counting backward, processing priority specified by task instructions cannot be maintained and gaze cueing effects from neutral faces are much reduced. However, such an account would also predict reduced gaze cueing effects for happy and angry faces under high cognitive load.

We suggest that in the gaze cueing task, whether emotional expression and gaze direction are processed even if task irrelevant, differentially depends on cognitive control mechanisms used to resolve the conflict between maintaining task priority (i.e., process the target and ignore the central face-cue) and processing priority for socially salient stimuli (i.e., be responsive to gaze as it may signal something in the environment but not to facial expression because it does not).

Finally, it should be mentioned that attentional bias and increased dwell time toward threat-related information are typical of anxiety disorders, e.g., [16, 65, 66] and that current accounts attribute these attentional biases to reduced prefrontal attentional control mechanisms to inhibit distractor processing e.g., [67]. The present findings provide direct evidence on the role of cognitive control mechanisms in reducing interference from angry distractor processing. To reiterate, cognitive control mechanisms are used to prevent interference from emotional faces in the gaze cueing task. When cognitive control mechanisms fail because taxed by a concurrent task, eye-gaze of happy and angry distractors exerts differential interference effects on information processing with angry faces increasing dwell time on the invalidly cued spatial location and happy faces affecting participants' preferences on the validly cued spatial location.

In conclusion, the present evidence helps clarifying the circumstances under which facial expressions modulate gaze cueing effects, and by doing so, provides an account for the complex findings present in the literature. Furthermore, it contributes to the literature on the role of cognitive control mechanisms to maintain processing priorities and shield performance from potential distractors interference e.g., [68], especially when there are high levels of competition/conflict between socially relevant but task-irrelevant stimuli e.g., [39].

## Supporting Information

S1 File Supporting Information Experiment 2. (DOCX)
