# Supplementary material for: Emotion Unchained: Facial Expression Modulates Gaze Cueing under Cognitive Load
Source: PLoS One. 2016 Dec 13;11(12):e0168111. doi: 10.1371/journal.pone.0168111 (PMC5154609; doi:10.1371/journal.pone.0168111)
Supplement: S1 File — (DOCX) [file pone.0168111.s001.docx]

To clarify whether the lack of gaze cueing effects for the neutral faces could be due to these stimuli not changing expression but only gaze direction or to load (even low cognitive load), in Experiment 2 participants performed the gaze cueing task only with neutral faces and without any concurrent load. To maintain the task as close as possible to that used in Experiment 1, participants were asked to rate their preference toward the target-objects in block 3.

Experiment 2

**Method**

**Participants**

Twenty-three participants, who had not participated in the previous experiment, took part in partial fulfilment of course credits (9 males, 11 females; age *M*= 23.3 years, *SD*= 4.0). They had normal or corrected to normal vision and were naïve to the experimental hypotheses. All participants gave their written informed consent, which was obtained according to the Declaration of Helsinki (1991). The experiment was in compliance with institutional guidelines and had received approval by the Department of Psychology Ethics Committee, Sapienza University.

**Materials, Apparatus, and Procedure**

Material, apparatus and procedure were as in experiment 1, with the only exception that participants did not concurrently perform any counting task and only trials with neutral faces were used. Participants completed 3 blocks of 72 trials, of which 32 valid, 32 invalid and 8 catch trials for a total of 216 trials.

**Data Analyses**

Gaze Cueing Task: Trials on which an error was made (1%) and with RTs faster than 120 ms or 2.5 SD above the mean (5%) were excluded from analyses. Mean RTs were computed and data were analysed with a repeated measures ANOVA with Gaze Cue (Valid *vs.* Invalid) as factor.

**Results**

ANOVA results for RTs showed a significant effect of Gaze Cue, *F*(1, 19)= 20.82, *p*< .000, partial η^2^= .523, with faster RTs when Gaze was a Valid Cue, *M*= 431 ms; *SE*= 22 compared to when it was an Invalid Cue, *M*= 544 ms; *SE*= 23.

ANOVA results for accuracy showed no differences between the two conditions, Gaze Cue, *F*(1, 19)= 3.85, *ns*.

Objects Preferences: ANOVA results for objects ratings in the last block showed no significant effect of Gaze Cue, *F*(1, 19)= 2.92, *ns*.

Therefore, these findings clearly show that the neutral faces used in Experiment 1 engender gaze cueing effects when participants perform the gaze cueing task without any concurrent cognitive load.
